# How sex chromosomes get trapped into nonrecombination

**DOI:** 10.1371/journal.pbio.3001718

**Published:** 2022-07-19

**Authors:** Jos Käfer

**Affiliations:** 1 Université de Lyon, Université Lyon 1, CNRS, Laboratoire de Biométrie et Biologie Evolutive UMR 5558, Villeurbanne, France; 2 Université de Montpellier, IRD, CIRAD, UMR Diversité Adaptation et Développement des plantes, Montpellier, France

## Abstract

We know that suppression of recombination leads to degeneration of Y chromosomes, but it has remained difficult to understand how features with such strong negative effects actually arise. This Primer explores a new model published in PLOS Biology that reveals how this could happen.

## Teaser

Suppression of recombination along the Y chromosome leads to its degeneration, so why does a process with such potentially deleterious consequences arise? In this issue of *PLOS Biology*, a new model reveals how and why this might be.

Sex chromosomes, like those in humans with a large X and small Y, have a long evolutionary history. Their difference is a dramatic consequence of the total absence of recombination of a portion of the Y chromosome, which is present in a single copy in males (and absent in females). The Y chromosome will therefore inevitably accumulate deleterious mutations, which cause it to degrade and lose genes, and maybe even to disappear [1]. While the effects of recombination suppression are well understood, it is not clear how it actually arises. Genes with sex-antagonistic effects could be responsible for this. An allele could, for example, have positive effects on male fitness and negative effects on female fitness; selection would favor linkage to the sex-determining gene on the Y. While this hypothesis is attractive, empirical support for it is weak or absent [2] and is sometimes even contradicted. For example, in fungi, where there is no sexual dimorphism, large nonrecombining regions have been found [3]. Jay and colleagues, in this issue of *PLOS Biology* [4], present a model for recombination suppression with minimal ingredients. They show that recombination suppression can arise because initially, fragments of chromosomes with fewer deleterious mutations than the population average exist, and these are thus favored. It’s somewhat ironic that this advantage rapidly turns into a trap with the precise contrary effect: the accumulation of deleterious mutations.

Recombination is essential for selection by creating new combinations of alleles and thus achieves the double task of keeping beneficial mutations while getting rid of the deleterious ones [5]. Without recombination, a beneficial allele that becomes fixed just drags all the deleterious mutations it is linked to along or it is lost if the cumulative effect of all deleterious mutations is too strong. The absence of recombination leads to a ratchet-like mechanism (“Muller’s ratchet”): As soon as there is no mutation-free strand of DNA anymore, it’s impossible to restore the original strand; in a next step, there will be at least 2 mutations on each strand, then 3, etc., in a never-ending process. With recombination, the average number of mutations on a strand of DNA will stabilize due to an equilibrium between mutation (which is inevitable) and selection.

In a population, stretches of DNA with more or less mutations exist. It is actually beneficial, on the short term, to preserve “lucky” stretches of DNA by preventing them from recombining with more mutation-laden counterparts. The most likely mechanism of recombination suppression is inversion of a part of the chromosome, which prevents DNA to pair properly during meiosis. As most deleterious mutations cause loss of function, they are largely recessive and have much stronger effects when homozygous. Thus, individuals carrying “lucky” inversions are favored as long as the frequency of the inversion in the population is low and homozygotes for it are very rare. However, as the frequency increases, homozygosity results in strong counterselection, which prevents such inversions to become fixed on autosomes.

**Fig 1:**
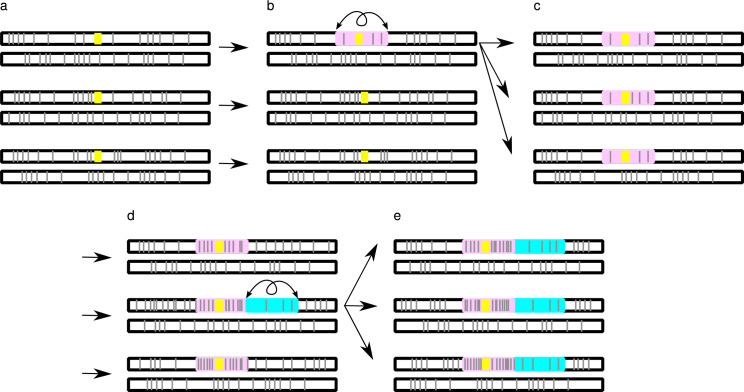
Schematic representation of the processes modeled by Jay and colleagues. Consider chromosomes with a sex-determining locus (yellow) and several deleterious mutations (gray bars). For the sake of simplicity, only 1 pair of chromosomes of 3 individuals of the heterogametic sex (XY males or ZW females) is shown. **(a)** The chromosomes all carry some deleterious mutations. **(b)** A “lucky” inversion (pink) captures fewer mutations than the population average and also includes the sex-determining locus. **(c)** This inversion gets fixed in the population and starts accumulating mutations. **(d)** As recombination is completely impossible in this region, it accumulates mutations and carries more than other regions in the genome. However, adjacent or (partially) overlapping, a new “lucky” inversion can arise (cyan). **(e)** This lucky inversion can become fixed as well, thereby creating a new evolutionary stratum.

However, as Jay and colleagues show, in the vicinity of a sex-determining locus, the result is quite different (Fig 1). Such loci are particular, because heterozygous individuals (XY) reproduce with homozygous individuals (XX), and the sex-determining locus on the Y never occurs as homozygote. Thus, a “lucky” inversion that encompasses this locus only experiences the positive effects related to heterozygote advantage and not the counterselection due to homozygosity. It can thus be fixed on the Y chromosome. Of course, it will experience the well-known disadvantages of recombination suppression, which lead inevitably to degeneration. But once the inversion is fixed, it’s too late: The only way out would be to restore recombination by the exact reversal of the inversion, which Jay and colleagues show to be very unlikely in most cases.

Surely, the selection of the inversion and the accumulation of deleterious mutations on it occur simultaneously, and the exact outcome (fixation or loss of the inversion) depends on the balance of these, and on chance. But, although the probability of fixation might be small, the result is irreversible. And it can occur repeatedly, leading to so-called evolutionary strata [6]. The model is applicable to other so-called “supergenes,” including ZW sex chromosomes and mating type loci.

Two other convincing models have been published recently, in which the suppression of recombination initially evolves without selection for sexual specialization. Jeffries and colleagues [7] consider that the accumulation of sequence divergence, possible when the recombination rates are lower around the sex-determining locus, can by itself reduce the probability of recombination, creating a positive feedback loop leading to the complete loss of recombination. This model is attractive because it doesn’t require inversions to happen, but it is not clear if the process can occur in reality. Lenormand and Roze [8] also considered fixation of recombination suppression (e.g., inversions) around a sex-determining locus due to less mutational load, as in the model of Jay and colleagues (Fig 1). However, they identify an entirely different mechanism that prevents the restoration of recombination: This happens through the evolution of dosage compensation, even in the absence of sex-specific optima for gene expression. The processes described in these models can occur together, and none forbid sex-antagonistic selection, which could thus also interfere.

There are important differences between nonrecombining regions; for example, in plant sex chromosomes, large nonrecombining regions can evolve quite quickly in some species, leading to very different X and Y chromosomes, while other, much older sex chromosomes have only small nonrecombining regions [9]. The reasons for these differences are not yet understood. Could these new models shed new light on this enigma? According to Jay and colleagues, selection during a haploid phase in the life cycle (plants), as well as the turnover of degenerated alleles in multiallelic systems (e.g., genetic self-incompatibility), could limit the expansion of the nonrecombining region. Furthermore, population size, the degree of outcrossing, and sexual dimorphism could play a role. Finally, inherently genomic features such as the distribution of recombination events along the genome, or the frequency and size of inversions, certainly influence the dynamics of recombination suppression, but these features have only been quantified in a few model species. A clear-cut answer is unlikely to emerge as the observed variation is large, even within clades, so the characterization of recombination suppression in more species is necessary to understand the interaction of the multiple factors involved.
